# The Progress of Medical Education

**Published:** 1898-11

**Authors:** 


### CORRESPONDENCE.

### THE PROGRESS OF MEDICAL EDUCATION.

Among the hopeful signs of the times in the medical world is the lessening of the number of colleges, by the consolidation of one or more reputable schools into one. This has been done in the North, West and South, and must result in good to the profession if the causes which seem to operate in producing these consolidations are properly taken advantage of.

To our mind there are many advantages to accrue both to the college and students by these consolidations.

In the first place, the *rivalry* always engendered, to a greater or less extent—by the location of two or more schools in the same city or even in the same geographical section—presents a strong temptation to receiving students not strictly in accordance with college or professional ethics. There is a strong temptation for the one or the other, or both, by some legerdemain of the conscience, which will protest against the charge of *palpable violation* of the law or published rules of their catalogues, to underbid for students. It has been too often the case that some special favor is granted or terms so made, by which the student in doubt as to which school he will select, feels that one has offered him better terms or greater inducements than the other. Now this is not healthy competition, and does harm both to the school and the student by compromising what is known to both as an ethical law and moral obligation, which is certain to breed animosity and strife between the professors and the students of the two schools, when in fact only a laudable emulation should exist.

In the second place, the old adage "in union there is strength," should be as applicable to college consolidation as to the ordinary affairs of life. It should bring a concentration of mind and energy to the great purpose in view—*i. e.*, the more thorough education of the medical student. Without the accomplishment of this end these consolidations will prove of no material benefit from an educational standpoint. If they are only for pecuniary gain, and no advance is made in the liberal and thorough education of the student, why, it is only a sham, a fraud, and should receive the reprobation it deserves.

Now that four years of study before graduation are required by most of the reputable colleges, there is time sufficient allowed the student (with a previously fair education, which all should have) to be GROUNDED in the elementary basis, the solid groundwork of the science of medicine upon which he must in the future rear the superstructure of the magnificent temple. He should spend two years or more under *competent* and *faithful* instructors in the dissecting-room and equally as long under the same conditions in the chemical laboratory. I have mentioned these two branches because they are the two in which the young graduate is most deficient when he goes forth to enter upon the active and practical duties of his profession. This is especially true of chemistry, because this branch cannot be learned from books, but only in the laboratory under *competent* and *painstaking* instructors. The instructor himself cannot be a competent teacher or demonstrator unless he spends a good part of his time in his laboratory previous to his lecture or has a competent assistant to prepare his experiments or make all analyses for him in the presence of the class, which should be taught practically to do the same.

Then, again, two years are not too long for the branch of materia medica and therapeutics, including botany. Not only the commercial and botanical history of all vegetable drugs, with their remedial properties, should be mastered, but the chemical composition of both organic and inorganic drugs likewise.

Pathological anatomy, with the practical use of the microscope, can be pursued with great advantage by consolidation and a four years' course.

"Everybody who is anybody" in the profession nowadays is a surgeon and gynecologist, and any graduate of a consolidated four years medical college who cannot remove an appendix or spay a woman had better abandon his profession and go to raising four-cent cotton.

AN OLD DOCTOR.

GRIFFIN, GA., October 12, 1898.

*Editor Atlanta Medical and Surgical Journal:*

The State Medical Examining Board met at the Capitol, in Atlanta, October 11th. Nine applicants applied for license, with the following result:

One—Long Island Medical College, 85 per cent. Passed.
One—Medical College of Alabama, 82 per cent. Passed.
One—University of Louisville, 71 per cent. Passed.
One—Baltimore Medical College, 87 per cent. Passed.
One—Atlanta Medical College, 75 per cent. Passed.
One—Medical College of Virginia, 82 per cent. Passed.
One—Chicago Medical College, 58 per cent. Failed.
One—Tufts Medical College, 66 per cent. Failed.
One—Louisville Medical College, 57 per cent. Failed.

I herewith enclose you copy of the questions.

Respectfully, E. R. ANTHONY,
Secretary.

